# Iowa State Medical Society

**Published:** 1869-07

**Authors:** A. G. Field

**Affiliations:** Secretary


### IOWA STATE MEDICAL SOCIETY.

### SYNOPSIS OF THE PROCEEDINGS OF THE SEVENTEENTH ANNUAL MEETING.

WEDNESDAY, *May 26th, 1869.*

The Iowa State Medical Society convened in the Hall of Good Templars, in the City of Des Moines, as per adjournment. The President, Dr. Philip Harvey, of Burlington, in the Chair. Prayer was offered by Rev. H. S. DeForest, who was present by invitation.

The minutes of the last annual meeting were read and approved.

Members present were Drs. J. C. Hughes, A. M. Carpenter, H. T. Cleaver, Keokuk; T. J. Caldwell, Adel; N. S. Smith,

Belle Plaine; Wm. Cornes, Tama City; Jas. Gambie, LeClaire; N. Steel, Fairfield; J. Williamson, S. B. Thrall, Ottumwa; W. F. Peck, E. H. Hazen, Davenport; H. L. Whitman, C. H. Rawson, J. O. Skinner, D. V. Cole, W. H. Ward, A. G. Field, Des Moines; J. W. Gustene, Panora; F. Loyd, Iowa City.

The President stated that the address which he had prepared was of a popular kind, and it had been suggested that it be delivered to a popular audience.

Dr. Hughes moved that the address be deferred until evening, and that a committee be appointed to procure a suitable room and give due notice.

After some discussion, the motion was amended so as to make the Hall of the Good Templars the place, and 2 o'clock P.M. the time, for the delivery of the address, and that all interested be cordially invited to be present; in which form the motion was unanimously adopted.

On motion, a committee of five was appointed to nominate officers for the ensuing year, consisting of Drs. W. F. Peck, E. H. Hazen, C. H. Rawson, N. Steel, A. M. Carpenter.

After some further discussion of topics relating to the business of the meeting, the Society, on motion, adjourned to meet at 2 P.M.

AFTERNOON SESSION.

The Society convened as per adjournment, the President in the Chair.

The annual address being in order, the President proceeded to deliver a carefully prepared and instructive address on the subject of "Climateology of Southern Iowa;" contrasting the climate of Iowa with that of other portions of the earth's surface, and furnishing explanations for some of the advantageous characteristics of this climate.

On motion of Dr. Field, a vote of thanks was tendered to Dr. Harvey for his very interesting and instructive address. Carried unanimously.

The Secretary was instructed to furnish to each of the daily papers published in this city a brief synopsis of the proceedings, such as might be of public interest.

The Editor of *The State Register* being present, requested, through the Secretary, a copy of the President's address for publication.

Dr. J. C. Hughes moved that the address be referred to the Publishing Committee, with permission to grant the request. Carried.

Dr. Steel, of Fairfield, read a report of a case of ascites, and operation for, which was received and referred to the Committee on Publication.

Drs. Peck and Hughes reported other interesting cases, which were followed by remarks by Drs. Carpenter, Hazen, Thrall, Harvey, and others. Reports received and referred to Committee on Publication.

The Board of Censors reported favorably upon the following named applicants for membership of the Society:—

Dr. J. F. Kennedy, Tipton; graduate of University of New York, 1858.

Dr. R. W. Pryce, Iowa City; Jefferson Medical College, Philadelphia, 1858.

Dr. John Bower, Guthrie Centre; Pennsylvania Medical College, Philadelphia, 1848.

Dr. George F. Jenkins, Keokuk; Missouri Medical College, 1866.

Dr. George P. Hannwalt, Des Moines; Medical Department of Georgetown College, D.C., 1864.

Dr. James H. Boucher, Iowa City; Jefferson Medical College, 1856.

Dr. James G. House, Independence; Columbian Medical College, D.C., 1841.

Dr. Charles W. Davis, Indianola; Rush Medical College, 1853.

Dr. J. B. Buchtel, Des Moines; Cleveland Medical College, 1857.

Dr. E. J. McGorrisk, Des Moines; St. Louis University, 1864.

Dr. John D. McCleary, Indianola; Keokuk Medical College.

Dr. J. Grimes, Wapello; Keokuk Medical College, 1865.

Dr. Wm. H. Baxter, Wilton; Keokuk Medical College, 1865.

Dr. David Beach, Des Moines; Keokuk Medical College, 1861.

Dr. J. F. Wakefield, of Greenbush; Rush Medical College, 1868.

H. C. Fitzgerald, Van Metre, Keokuk Medical College, 1869.

Upon motion, the report was received, and each applicant duly elected a member of the Society.

Dr. Peck moved that when we adjourn, we adjourn to meet at 8 o'clock this evening. Carried.

Dr. Peck read a report of some especial cases of surgery, which was received and referred to the Committee on Publication.

The President stated that it was necessary to take some action in relation to the death of the late Dr. Whinnery, of Fort Madison.

On motion, Drs Carpenter, Thrall, and Peck were appointed to draft resolutions expressive of the sense of the Society on the death of the deceased.

The report of Standing Committees being in order, the Committee on Surgery read a report, which was received and referred to the Publishing Committee.

The Committee on Epidemics read a report, which was received and referred to the Publishing Committee.

The Committee on Puerperal Diseases offered a verbal report as a substitute for a written one, which the Chairman stated had been prepared and left at home.

On motion, the Committee were requested to forward the report to the Publishing Committee.

On motion of Dr. Peck, the subject of the use of carbolic acid was designated as a subject for discussion this evening.

On motion, the Society adjourned to meet at 8 o'clock P.M.

EVENING SESSION.

The Society met as per adjournment.

The President announced the subject for discussion, and remarks were made by Drs. Hazen, Carpenter, Rawson, Jenkins, Hawley, and others.

Dr. Carpenter, Chairman on Necrology, offered the following, which was received and unanimously adopted:—

Whereas, This Society has heard with deep sorrow of the death of Dr. Edward Whinnery, of Fort Madison, late Vice-President of this Society, and for many years one of the most zealous workers in the cause of science, and in the diffusion of moral influences among its members; therefore—

*Resolved,* That in his sudden calling off we recognize the omnipotence of the divine will of Him who giveth and taketh away; and may we emulate the example of our late worthy Vice-President.

*Resolved,* That in the death of Dr. Whinnery this Society deeply feels the loss of one whose pleasure it was to labor for its growth and usefulness.

*Resolved,* That as a tribute of respect to his memory, these resolutions shall be spread upon the records of the Society, and a copy of the same be transmitted to the family of the deceased, and that they be published in the *Iowa Medical Journal.*

On motion, a committee was appointed to report upon the value of an honorary degree as a claim to membership—Drs. Floyd, Peck, and Whitman, Committee.

The Committee on Drafts for Bills and Order of Business, appointed at the last annual meeting, of which Dr. W. F. Peck is Chairman, was, upon motion, continued.

Adjourned to meet at 8 o'clock to-morrow morning.

MORNING SESSION.

The Society met as per adjournment. The President in the Chair. The minutes of the proceedings of yesterday were read and approved.

On motion, Dr. Kennedy was requested to read reports of two cases of gun-shot wounds, which were received and referred to the Committee on Publication.

The Committee on the Uses and Abuses of Mercury offered a report which was received and referred to the Committee on Publication.

The Committee on Special Surgery read a report, which was received and referred to the Committee on Publication.

The Committee on Medical Topography and Meteorology made partial report, and the Committee was continued.

It was announced that Adjutant-General Baker had invited the Society to visit the Arsenal at 10 o'clock to-day; whereupon the following resolution was adopted unanimously:—

*Resolved*, That the thanks of this Society are hereby tendered to Gen. N. B. Baker for his kind invitation, but that acceptance thereof is incompatible, at this hour, with the business of the Society.

The Committee on Nominations for officers for the ensuing year offered the following report:

*President*—Dr. S. B. Thrall, of Ottumwa; *Vice-President*—Dr. N. Steele, of Fairfield; *Secretary*—Dr. A. G. Field, of Des Moines; *Treasurer*—H. L. Whitman, Des Moines; *Corresponding Secretary*—Dr. E. H. Hazen, of Davenport. *For Censors*—Drs. J. Williamson, of Ottumwa; M. A. Dashielle, of Hartford; Wm. Watson, Dubuque; James Gamble, Le Claire; George F. Jenkins, Sandusky; and David Beach, Des Moines. *For Trustees*—First District—Dr. A. M. Carpenter, Keokuk; Second District—Dr. W. F. Peck, of Davenport; Third District—Dr. J. C. Lay, of Dubuque; Fourth District—Dr. Wm. Voght, of Iowa City; Fifth District—Dr. C. H. Rawson, of Des Moines; Sixth District—Dr. N. S. Smith, of Belle Plaine.

*Publishing Committee*—Drs. H. L. Whitman, and A. G. Field, Des Moines; J. Williamson, Ottumwa; and A. M. Carpenter, Keokuk.

The report was received and unanimously adopted.

Adjourned to meet at 2 P.M.

AFTERNOON SESSION. 2 O'CLOCK P.M.

The Society convened as per adjournment.

Drs. Hughes and Caldwell were appointed to conduct the newly elected President to the Chair, who, on taking it, made a few remarks, thanking the Society for the honor conferred.

On motion, the Committee on Publication was given discretionary powers as to the discharge of its duties.

The report of the Publishing Committee was read and adopted.

The report of the Treasurer was received and adopted.

The financial report of the Secretary was received and adopted.

On motion, the Standing Committees are to consist of one each.

On motion, the Society adjourned to meet on the third Wednesday of February, 1870, in the City of Des Moines.

A. G. FIELD, *Secretary.*

